# Oral Electricity and the New Departure

**Published:** 1881-09

**Authors:** John J. R. Patrick


AMERICAN JOURNAL

OF

DENTAL SCIENCE.

Vol. XV. THIRD SERIES—SEPTEMBER, 1881. No. 5.

### ARTICLE I.

### "*Oral Electricity and the New Departure.*"

BY DR. JOHN J. R. PATRICK.

In questions of physics, or the laws governing material substances, mere opinion is often confounded with demonstration. I believe that most men are honest in their statements concerning their experience, their experiments, and their observations; but these must be capable of verification and demonstration, otherwise we should be in the condition of the man who resolved to regulate his watch by the town-clock, but would not inquire whether the town-clock was right or not. Outside of the known laws which govern the properties of matter, all becomes conjecture; and as there is no limit to the possible conjectures one may make regarding things beyond the power of demonstration, there can be no end to the disagreements that will arise if we permit ourselves to embark on this troubled sea of dignified guess-work.

The materials used for filling teeth cannot be intelligently discussed independently of the tooth-substance and its surroundings. Every element that enters into the composition of the living body, and every substance that is likely to come in contact with the material used for filling the teeth, the chemical affinity of the several substances present in the mouth, and the energy or force set free by molecular change, must all be considered before a scientific deduction can be established.

Opinions and theories of men, based on their own experience and observation, especially when such experience relates to scientific matters or manipulative skill, include so many undecided questions, that it is not only unscientific, but dangerous, and even frivolous to hastily adopt them. The frequent failures in filling teeth with gold by the most conscientious operators—men who spare no pains to perform their work to the utmost of their ability—are too apt to lead them to seek the cause of failure outside of themselves; for no man who honestly does his utmost is willing to suspect a lack of manipulative skill as the cause of his failure. No man feels composed under the imputation of a want of skill in his profession. If he is an imaginative man, he at once seeks redress and an explanation of his failures in a wilderness of speculation, in which there is no definite coherence. Hypothetic currents of electricity are mixed up with incompatibility of tooth-substance, chemical interaction and filling material. Imagination unconsciously takes the place of reason, the inventive faculty is exercised in the interests of previous expectation, and emotional experiments are prosecuted with all the outward appearance of scientific accuracy—for scientific instruments are used (or rather abused) with automatic activity, regardless of the laws of exact association, and in the interests of the dominant idea. There are so many obscure phenomena connected with electricity, so much of its action is undecided, and not reduced to law, that it offers superior inducements to persons of a marvelous tendency to revel in its mysteries, and to float in confident security, like the atoms of Epicurus, in a void.

There are other causes that have been equally as fruitful as defective manipulation in creating a belief in the superiority of plastic filling.

1st. The continued appeals on the part of patients for inexpensive work. For instance, it is frequently the case that a well-dressed man, wearing diamond jewelry, will request his dentist to use the cheapest possible material; and a majority of women will spend more money in one year with their milliners than they will with their dentists in ten years, and still feel the tax of the dentist the heavier of the two.

2d. The ease of the patient and operator in the use of plastic materials.

3d. The more profitable employment in a monetary point of view to the operator.

4th. The deep interest shown by the champion and promulgators of this old system revived in putting compounds of their own manufacture upon the market.

Prof. J. Foster Flagg has given to the world his pamphlets on the "Basal Principles of the New Departure," and declares that he is but the mouth-piece of Prof. Henry S. Chase and Dr. S. B. Palmer, and fully indorses the creed of these two gentlemen, that "The electro-chemical theory of the action of filling material and tooth-substance, and the incompatibility of gold with 'Dentos,' make gold the worst material to use for saving teeth."

Prof. Flagg further states in his pamphlet that with the assistance of Prof. Morton, of Hoboken; Prof. Snyder, of Philadelphia; Messrs. Eckfelt and Dubois, assayers of the Philadelphia Mint, together with Drs. Palmer and Chase, dentists, he has "carefully and scientifically investigated, with the best instruments and apparatus the country has produced, every line of experiment which professed to show a current, which they believed to be eliminated by contact of metal with tooth bone, and found that such current had never been shown, much less measured." So that Prof. Flagg falls back upon belief and his own acknowledged failures to preserve teeth with gold for the hypothesis of electric currents destroying tooth-bone when gold is employed in filling teeth.

Prof. Flagg betrays the influence of some of the inducements I have enumerated for using plastic fillings in preference to gold, when he says that "teeth are safer in the hands of the majority of practitioners turned out from our schools with a plastic material for filling than with gold." This is a humiliating confession, and so much the worse for our professors and our schools; for the dentist who cannot manipulate gold, either for plate or for filling, must take a back seat ; he has no business in the profession. Notwithstanding the failure to demonstrate the electric current, Dr. Flagg constantly refers his readers to the two dental electricians, Drs. Palmer and Chase, and informs us that letters by the score passed between Syracuse, St. Louis and Philadelphia on the subject, and that finally these three gentlemen were ready to promulgate the doctrines of the New Departure, or, more properly speaking, they were ready to present to the profession some new honey that they had abstracted from old hives. In reviewing the published experiments of Dr. Chase, which are indorsed by his co-laborers, I find them so full of fallacies, contradictions and misconstructions of physical law, that a few instances will suffice to render his whole line of experiments worthless. On pages 201 and 202 of the *Missouri Dental Journal* for the year 1876, the following sentences occur:

"1st. The oral cavity may be compared in some sense to a single cell of a voltaic battery.

"2d. Where the teeth are all sound and the oral fluids are in a perfectly physiologicial condition, the mouth is not a voltaic battery.

"3d. There is probably more or less electricity evolved in the ordinary mouth nearly all the time, more especially if there is a tooth plugged with any metal whatever."

Here are three equivocal statements, if not contradictions. The first compares the mouth to a voltaic battery, the second denies it, and the third sustains the first proposition.

On pages 202 and 203 the doctor states a well-established law in electro-metallurgy,—that "The electric current passes from the positive element to the negative one, and that element is positive which is most easily acted upon by the fluid in which the elements are immersed; and that element is negative which is less acted upon." In other words, the positive element is that metal which is dissolved, and the negative element is that metal or substance upon which the metal is being deposited by the influence of the electric current, electricity simply being the carrier of the metal from the positive to the negative pole, as in the electrotype process. On the same page (203) the doctor says that "*dentos* is a bad conductor of electricity, and therefore it is a negative element in the "*oral battery.*"

I reply that a tooth is a good conductor of electricity, since it is permeated by the fluids of the tubuli. For proof of this assertion, grasp one of the poles of a battery and bring the other pole in contact with the teeth, which will readily determine this question. If "dentos" were a negative element in the "oral battery" and the filling material positive, by all the laws of electro-metallurgy the filling material would go to the "dentos" (the place where it usually is needed,) not the "dentos" to the filling material. In other words, the metal filling being positive must waste away and be carried by the current to the "dentos" and be deposited upon it either in the form of salts or of metal, and the dentine will remain intact.

The whole of page 204 is devoted to contradicting his former statements, and to promulgating a few electrical impossibilities. One instance will suffice: "The electrical action between two substances is none the less on account of the resistance offered to the passage of the current."

Now, it is a well-established fact among electro-metallurgists that the negative element is only considered as acting the part of a conductor, and resistance to the passage of a current stops the action.

On page 205 he says, "A piece of muscle impacted between two teeth will produce considerable of a current, which is usually so annoying that removal of the substance seems an immediate necessity to the individual."

Of course, when an electrician has the cause to investigate, this annoyance could not be produced by the simple effect of mechanical pressure.

On page 208 the doctor recommends the use of tin and gold, and describes the method of using them: "But the better method to use tin and gold in the same cavity is to employ cylinders of tin only, and of gold only, always keeping the tin next to the walls of the cavity and using only gold in the centre of the plug." I will simply state that the interposing of tin between gold and the substance of the tooth as a protector of the dentine from the action of the gold (if such action existed) would not affect the polarity in the least; the tin would act merely as a conductor. But if I wished to form a little cell or electric combination in the mouth by the simple contact of two metals, I could hardly devise a better method, for gold being one of the most negative metals, a feeble action might take place between the two.

Currents produced by contact alone are always extremely weak. A much stronger current would take place between an amalgam of zinc and one of silver, placed in the same position, for, although silver is not as negative a metal as gold, it possesses the highest conductive power of all metals. The tooth-substance would not be a factor in the case, for we all know that tooth-substance is composed of salts, which always impede electric currents, the gelatine and water being the only material in the tooth that could act as a conductor. A desiccated tooth would be a complete insulator.

Gold is not attacked by any single acid (except selenic,) does not tarnish in dry or moist atmosphere at any temperature, and is not affected by sulphur, iodine or phosphorous. Tin, on the contrary, tarnishes or oxidizes slowly in moist atmosphere. *Its oxides combine with vegetable acids*, and its salts in solution will attack and combine chemically with the substance of the teeth. Its power to chemically unite with tooth-substance is only equaled by mercury and silver. When new compounds are formed by the chemical affinity of elementary bodies, and change of molecules takes place, or when compounds are recompounded by the same agency, organisms may be destroyed or built up. In either case passive or active electricity may be evolved. This law of chemical affinity is beautifully illustrated in the incubation of an egg. It is well known that the body of an egg contains neither phosphoric acid nor lime, but both these substances exist in the shell, which becomes thinner and thinner during the whole time of incubation, till the living embryon appropriates a sufficient quantity for the formation of its bones; part of the albumen combines with the shell for this purpose, and another portion forms the feathers.

It is also remarkable that though phosphate of lime is always present in the urine of adults, it is not found in the urine of infants; the rapid formation of bones in the first periods of life appropriates all of this salt eliminated. So we can rest content, if the phosphate of lime is present in the urine, that the supply is greater than the demand for it, in forming tooth-substance.

What Dr. Chase and his co-laborers wish to prove is, that gold, from its power to resist chemical action, is to be considered the worst material for filling teeth, because it is one of the most negative of metals,—that is, that the dentos is dissolved and goes over to the negative metal; but by his own confession his instruments do not indicate that dentos is a positive element in his oral battery. Dr. W. C. Barrett, of Buffalo, New York, in his very able review of the so-called "New Departure," very successfully quotes Dr. Palmer against himself. In a paper read before the State Dental Society, of New York, in 1874, Dr. Palmer says, "A porous tooth containing an amalgam plug has in it the elements of a minute but intense *battery* capable of decomposing not only the plug but the tooth around it." This early statement of Dr. Palmer proposes an impossibility, for gold, silver, platinum and tin, fused together and then reduced to filings and afterwards amalgamated with 60 per cent. of mercury, *would not* and *could not* polarize to form an electric combination, much less a "battery." The molecules of each separate metal would be positive and negative to each other in such a confusion of infinite quantities that they would neutralize each other's action. I know of no means of forming an electric combination in the mouth except by filling a superior tooth with a zinc amalgam, and an inferior opposite one with a silver amalgam, so that they would come in contact. Then I admit that a circuit would be formed through the moist tissues of the mouth from the zinc to the silver, and in case of an acid condition of the fluids of the mouth, electric excitement would take place every time the two metals came in contact;—if the fluids were neutral, electric excitement would take place feebly.

That electric currents should be generated in the mouth, and that when present they should be destructive of bony tissue, so far from being true from a scientific point of view, is not, I think, even theoretically possible, for the following reasons:

1st. The sense of taste is an extremely delicate test of an electric current; signals may be tasted which even the most delicate galvanometer will fail to detect.

2d. Very strong currents of electricity have been used daily for months and years as a therapeutic agent in cases of paralysis, and it is in constant use as a stimulant to tetanize dormant muscle without being in the least destructive to bony tissue.

It is not electricity that does the mischief; it is defective filling with gold and a change of molecules in other materials used for filling teeth, and this takes place when no current could be perceptible. Diffused electricity may take place on the decomposition of any substance, but it must be remembered that in this instance electricity is the result, and not the cause, of molecular change.

In the June (1877) number of the *Missouri Dental Journal,* on page 223, Dr. Chase sums up the results of his experiments and observations, and embodies them in the following sentences: "Gold is not oxidizable in the mouth, and in itself may be said to be permanent, but the tooth suffers more from that fact. *It is the tooth-substance* that does not last; the metals of which plugs are made *last* long enough. Their slow surface chemical disintegration is *desirable* in an acid condition of the mouth. A plug that will not do this is a greater enemy to dentos than one that will." These sentences need no comment.

In the *Journal* of July 15, of the same year, the doctor gives freely the results of his experiments in dental alloys, and publishes three formulas. The first formula contains equal parts of gold, silver, and tin, amalgamated with 80 per cent. of mercury, or, if 20 parts of tin filings are added, 60 per cent., which makes 146⅔ parts of tin, silver, and mercury, and 33⅓ parts of gold. This amalgam, while it contains so small a proportion of gold, is far richer in that metal than any alloy in the market, for where platinum is used it only takes the place of gold or divides the honors with that metal.

Now, why use gold at all? If gold is incompatible with tooth-substance, so is platinum. Why use these expensive metals in such connection? Are there no fears that this seductive and dangerous gold will corrupt the virtues of the other four metals? Is it not a short-sighted liberality to use it in such connection when it is useless? But if it be dangerous to the integrity of tooth-substance, is it not culpable?

When Thomas Fletcher produced the platinum and gold alloy, it was an improvement on Dr. Townsend's amalgam of tin and silver, as much as Dr. Townsend's amalgam was an improvement on the zinc and tin amalgams in use forty years ago; and I can assure you it was no spasmodic fit of emotional generosity that induced Fletcher to add the gold and platinum to the silver and tin of Townsend and smelt them together, producing a more intimate connection of their particles than could have been obtained in the filings of the separate metals. The wide difference of expansion and contraction of the several metals under thermal influence is to a trifling extent compensated thereby. This was his object, and this is why test-tubes were introduced. The elements that are constantly present in the body, that unite directly with silver, tin, and mercury, were no consideration with Thomas Fletcher, and, as far as this controversy has gone, I am not aware that they have been introduced by any one. I, therefore, with the tenderest regard for electricity, beg leave to present for your consideration, sulphur, iodine, and phosporous, a trinity of elements which have a great attachment for silver, mercury, and tin. But more especially do I recommend them to the attention of the advocates of this new doctrine.

### SULPHUR.

There is no other element so widely distributed in the animal, vegetable, and mineral kingdoms as sulphur. Nature employs it in most of her operations. She presents it under many forms among fossils; charges with it the water known as sulphurous; mineralizes with it most of the metals forming iron pyrites, galena, cinnabar blende, and silver glance; causes it to pass into the vegetable and animal fibres, and forms an infinite number of combinations with the earths. Sulphur is present in horse-radish, cresses, and many other vegetables, and is a constituent of the volatile oils of mustard, garlic, and assafœtida, all of albumen and other proteids. It is always evolved, in combination with hydrogen, from animal substances during their putrefaction; and the change which silver undergoes when immersed in mustard or an egg shows the presence of sulphur, and the strong affinity it has for that metal. When taken into the system with food, or exhibited as a medicine, it penetrates to the extremities of the most minute vessels, and impregnates all the secretions. It shows a tendency to unite with mercury equally as strong as its affinity for metallic silver, for there are instances on record of persons having been subjected to mercurial medicine for a short time, and upon the outward application of sulphur ointment for a few hours, the spot where the ointment was applied became quite black. This was occasioned by the mercury exuding through the pores of the skin to unite with the sulphur, in consequence of its affinity for that substance, and a true ethiops-mineral was formed. A beautiful illustration of this principle is shown in one process of parting gold and silver: The silver being in excess, the two metals are melted and rolled into plates; then a layer of sulphur and a plate are alternately disposed, whereupon the sulphur unites with the silver, forming a sulphuret of silver in the form of a black powder, which is washed out, leaving the gold in a spongy condition, but pure. Sulphur does not unite with metallic gold or platinum, but it will form an auric sulphide when united with the cyanide of gold.

The constant presence of sulphur in the system, and the intense chemical energy of sulphur for silver and mercury, render these metals unfit for the purpose of saving teeth. The small amount of gold and platinum present in Fletcher's or any other alloy, gives no protection to silver or mercury from the invasion of sulphur. Sulphuret of silver and of mercury is formed in the cavity of the tooth in which an amalgam filling is placed, for the reason that the fluids in the tooth-substance convey the sulphur to the silver. Any amalgam filling removed will be found to be black where it comes it contact with tooth-substance, I care not how the amalgam may have stood the test for leakage in glass tubes before using. When we reflect on the complicated structure of a tooth, and consider the facts that a constant circulation is sustained as long as vitality remains, and that even in a dead tooth the same canals permit the fluids of the body to pass through its substance, the pleasing thought of a moisture-tight filling, that will not allow any moisture between itself and the bony walls which surround it, becomes a mere phantom. Oxygen has been accused of darkening amalgam and tin fillings on the outside. It does its share where the fillings are exposed to the atmosphere, but sulphur attacks them all over. Sulphur will sever its connection with rubber after it has been vulcanized to unite with silver, if silver is placed in contact.

### IODINE.

Iodine, next to sulphur, is one of the most widely diffused of elementary bodies. It vaporizes at the ordinary temperatures; it is found in some minerals combined with mercury and silver; unites readily with the different salts, but enters into combination directly with metallic mercury, silver, tin, copper, and zinc, forming the iodides of these metals. Aside from its natural presence in the system, it is used largely as a therapeutic agent (stimulant, absorbent, emmenagogue, and alterative.) From its known affinity for mercury, it is not used in collutories for relieving mercurial sore mouth; and in cases of chronic mercurial poisoning the administration of iodine furnishes the most effective means of eliminating mercury from the system, thereby curing paralysis, neuralgia, and other symptoms of poisoning from this metal, for all soluble salts of mercury are poisonous.

### PHOSPHOROUS.

This non metallic element is widely diffused; it enters largely into combination with the oxide of calcium (lime) and magnesium in the formation of bone; and in the dentine of the human teeth the phosphate of lime and magnesia constitute 63 per cent., and in the enamel 86 per cent. Phosphorous unites readily with metallic salts, and unites slowly with metallic silver, mercury, tin, copper, and zinc, but it will not unite with metallic gold or platinum.

### CHLORIDE OF SODIUM.

This compound of muriatic acid and sodium, when reduced to dryness, forming the chloride of sodium, is found native in extensive beds, and enters largely into all organized bodies, and is essential to the digestion of food. A great deal has been said and written in regard to the combination of this salt with the mercury contained in an amalgam filling, forming the chloride of mercury (calomel,) and the corrosive chloride of mercury (corrosive sub.) I confess that I do not see how such a compound can be produced in the human mouth, for the sulphate of mercury must first be formed with sulphuric acid, then boiled to a dry powder, the salt added afterwards, and the two rubbed together, and then sublimed with a gradual heat, when the vapors of perchloride of mercury arising are condensed in the cool part of the apparatus, which vapors are the chloride of mercury. A very simple or mild chloride of mercury can be formed by rubbing metallic mercury with salt, but such a combination would be mechanical, and, I think, harmless. If, however, the sulphuret of mercury had formed around large amalgam fillings, and had combined with the salt in the system, or with that taken into the mouth, there would certainly be produced a chloride of mercury of a very mild character; as all soluble salts of mercury are poisonous, and it being a well-established fact that some persons are more susceptible to the influence of mercury than others, if there were no other reasons for discarding the use of this metal in the mouth, than the fear of this combination, this alone should be sufficient to throw it out from the list of filling materials.

In the selection of filling materials, to secure a good result it is indispensable that the filling should fit the walls of the cavity perfectly, and that it should exclude completely all those external agencies which induce decay; that it should be solid and undivided in all its parts, fitting it to resist all pressure likely to be brought to bear against it; and, finally, that it should resist to the fullest extent chemical action, thermal influence and attrition.

So long as we are confined to the use of metals for filling material, there is no metal or combination of metals or substances that has yet been found that can take the place of gold for that purpose. It possesses more of the valuable properties of the force of cohesion (hardness, elasticity, malleability, ductility, etc.) than any other metal; it is capable of receiving and retaining additional powers of cohesion by the application of coercive force; and when combined with small quantities of its sister-metal, platinum, its hardness and elasticity are greatly enchanced by the joint action of adhesion and cohesion; in fact, the force of cohesion in gold is so powerful, that at ordinary temperatures it will unite under coercive force almost equally as well as iron or platinum under the influence of heat. Like platinum, it is not influenced by any single acid or alkali; it does not tarnish in moist or dry atmosphere at any temperature, and is not affected by sulphur, iodine, or phosphorous.

### COHESION OF AMALGAMS.

All metals lose their natural coherence when alloyed, and when amalgamated with mercury the standard of cohesion is further reduced. Silver and mercury do not oxidize in the atmosphere at ordinary temperatures, but when the silver is amalgamated with mercury the silver oxidizes, owing to its having lost much of its cohesive force. Platinum and mercury do not unite,—the hardest metal to fuse and the metal which is in a fused state at ordinary temperature have no sympathy for each other. Copper will not unite with mercury, but will unite with the nitrate of mercury, and the combination will then receive additional quantities of the metal mercury.

On contact, gold and tin, either separately or combined, unite mercury perfectly, and give up their cohesive properties in their combination with the mercury; for this reason the amalgam does not harden, and the gold and tin lose all their individuality in the combination.

Silver, and zinc, either separately or combined, unite readily with mercury; they lose much of their cohesive force, expand in hardening, destroying the fluidity of the mercury, and losing all their own peculiar properties.

### EXPANSION AND CONTRACTION OF METALS.

The superficial expansion of a metal is equal to twice its linear expansion, and the cubical expansion to three times the linear. The metals used by the dental profession are the following, the most expansible standing first in order: mercury, zinc, tin, silver, copper, gold, platinum.

Mercury is so expansible that it is classed among the liquids, and vapor arises from it even at the freezing point of water in sufficient quantity to whiten gold-leaf; the temperature of the human body (98° F.) is sufficient to overcome the whole of its cohesive force. Thus mercury, tin, and silver, forming nearly the whole body of amalgam fillings, must be measured by their cubical expansion under the influence of heat, and a corresponding contraction under the influence of cold.

### CONDUCTION.

The thermal conductivity of some of the metals in use by the dentist stands in the following order: silver, copper, gold, tin, platinum, and bismuth. The resistance offered by mercury to the conduction of heat is far less than that of water; it, however, offers more resistance to the conduction of heat than bismuth, which stands the lowest in point of conduction in the table of the conductivity of metals. Mercury is therefore an intermediate body, occupying a position between liquids and solids in its power of conduction, as well as in its power of coherence.

New and original research requires a singular independence of mind, and, unfortunately for the cause of progress, such minds are at all times rare. The electro-chemical theory for the destruction of tooth substance in contact with gold only lacks the facts to sustain it, but when men need facts to sustain a favorite or plausible theory, they are not long in seeing the facts required; everything becomes clear in the uncertainty; a little experiment, a little straining of the eyes, and all is seen that they believe they ought to see. And thus they sail on an unknown ocean, trusting to hypothetical charts, to compasses of their own invention, in which the essential feature is the omission of that magnet which can alone give security.

Yet for ages this has been the only seamanship deemed necessary or practical in the history of medicine and its many specialties. And how many still trust to it implicitly! Thanks to the inroads that physical science has made on our domain, the dawn of a better day is fast approaching, and the next generation must witness disclosures not less startling than those which have electrified the present day. In the meantime what we want is a careful classification of existing facts (physics, not metaphysics,) their exact relation to each other, the great aim being to discover a genuine sequence of phenomena traced link by link to a definite cause.

The opinions advanced by Professors Flagg and Chase, and Dr. Palmer, are acquiesced in, either actively or passively, by many of those with whom they are working, as well as by others who are but slightly acquainted with the subject themselves, but who very naturally look up to the professors in their scientific character as authorities in all such matters. Were these simply theoretic opinions, they might very safely be left to the ordinary action of that silent criticism which quietly hands over to oblivion so many errors. But when we find them taken up by active, energetic men, and used as an important portion of their working machinery to increase the sale of mercurial compounds for filling material, they must be met in a formal and emphatic manner; and this becomes all the more necessary when we find them countenanced by writers in the same profession who have little, if any, *monetary* feeling in the matter, but who have simply been misled by superficial considerations.

RESUME.

In recapitulating some of the observations recorded in this essay, I desire to call attention to the following propositions: A voltaic battery can not exist except in the manner directed by Professor Volta, the inventor or discoverer. It consists of an equal number of pieces of zinc, silver, and pasteboard ; the pasteboard pieces are soaked in a solution of chloride of sodium, and then are piled with the metals in the following manner : zinc, silver, pasteboard, and so on, in the same order, the uppermost plate being of silver, and the undermost plate of zinc. These exterior plates, to each of which a wire is attached, form the terminals or poles of the pile. This was the first form of those instruments now so well known by the general name, "galvanic battery." The word battery is not applicable to any arrangement of elements present in the mouth. An electric battery is a collection of distinct cells, each cell so arranged in its elements as to secure an electric current and each cell so joined to the others as to secure an unity of action in the whole, thus forming a battery.

The methods of construction of batteries for the generation of electricity are various and peculiar, both in regard to the material used and the arrangement. Thus we have a Smee's, a Grove's, and a Daniell's battery, each bearing the name of its inventor; and when "Dentos and Gold" shall be proved to form an electrical combination, and be proved to be a battery, *it* may also bear the name or names of the inventors.

If vapor rises from mercury at the freezing point of water in sufficient quantity to whiten gold-leaf, an amalgam filling subjected to the heat of the human body (98° F.) must gradually part with its mercury by vaporization. This can be demonstrated by selecting a few old amalgam fillings, and, after accurately weighing them, submitting them to a heat sufficient to evaporate mercury. This being done, let the mass cool and again weigh, when it will be found to weigh as much as it did before heating. As a further test of the divisibility of mercury under the influence of heat, take one hundred parts of Fletcher's alloy, add sixty parts by weight of mercury, and allow it to harden ; then submit it to a sufficient heat to vaporize the mercury, and weigh the residue, and there will be found one hundred parts,—the mercury having disappeared.

Therefore, when an individual, either by bad counsel or a false economy, has been subjected to mercurial or amalgam treatment for diseased teeth, we find that fifty parts of the mercury out of the sixty used in forming the amalgam are vaporized by the heat of the body in a few years, and taken into the system in small but regular quantities,—the most potent manner of administering mercury for a constitutional effect. Should not this be sufficient to induce every conscientious practitioner to discard all amalgams from the list of filling materials, and be the means of inducing others to be less presumptious in their speculations, and more honorable and resolute in their practice.—*Dental Register.*

